# Supplementary material for: The Comparative Analysis of Antiviral Activity of Native and Modified Fucoidans from Brown Algae Fucus evanescens In Vitro and In Vivo
Source: Mar Drugs. 2020 Apr 22;18(4):224. doi: 10.3390/md18040224 (PMC7230360; doi:10.3390/md18040224)
Supplement: Supplementary file 1 [file marinedrugs-18-00224-s001.pdf]

Supplementary material

The Comparative Analysis of Antiviral Activity of Native and Modified Fucoidans from Brown Algae *Fucus evanescens In Vitro* and *In Vivo*

Natalya V. Krylova [1,*], Svetlana P. Ermakova [2], Vyacheslav F. Lavrov [3], Irina A. Leneva [3], Galina G. Kompanets [1], Olga V. Iunikhina [1], Marina N. Nosik [3], Linna K. Ebralidze [3], Irina N. Falynskova [3], Artem S. Silchenko [2] and Tatyana S. Zaporozhets [1]

[1] G.P. Somov Institute of Epidemiology and Microbiology, Vladivostok 690087, Russia; galkom1965@gmail.com (G.G.K.); olga_iun@inbox.ru (O.V.I.); niiem_vl@mail.ru (T.S.Z.)
[2] G.B. Elyakov Pacific Institute of Bioorganic Chemistry, Vladivostok 690022, Russia; swetlana_e@mail.ru (S.P.E.); artem.silchencko@yandex.ru (A.S.S.)
[3] I.I. Mechnikov Research Institute of Vaccines and Sera, Moscow 105064, Russia; v.f.lavrov@inbox.ru (V.F.L.); wnyfd385@yandex.ru (I.A.L.); mnossik@yandex.ru (M.N.N.); lina.lidze@gmail.com (L.K.E.); falynskova@mail.ru (I.N.F.)
* Correspondence: krylovanatalya@gmail.com; Tel.: +7-9084-486-423;

A

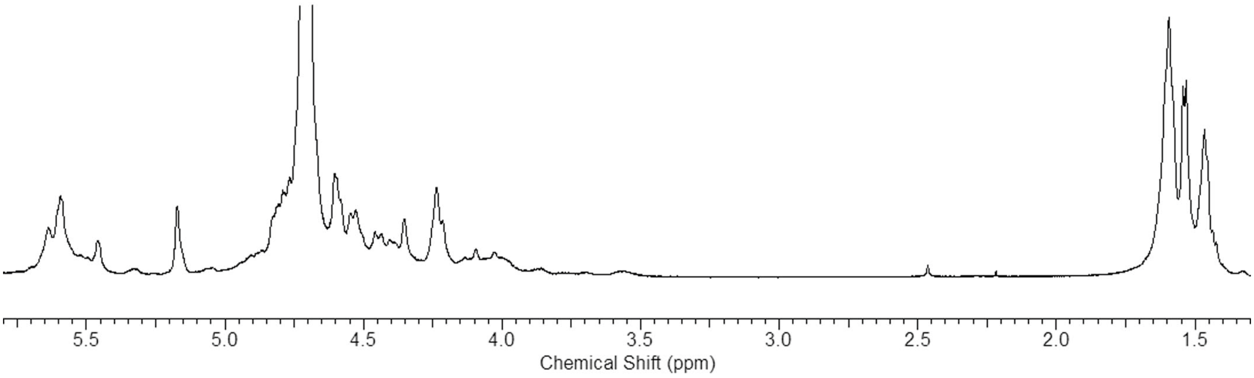

B

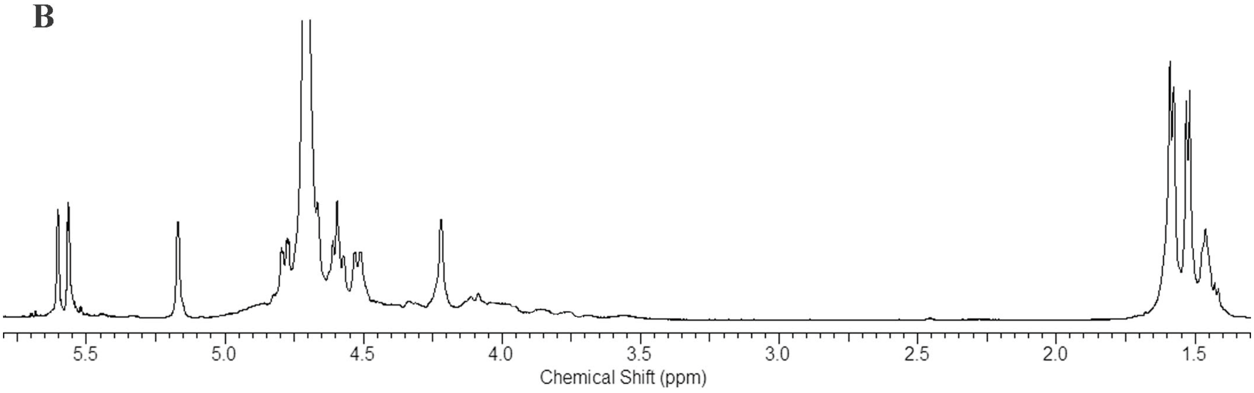

Figure S1. ^{1}H NMR spectra of native fucoidan FeF from F. evanescence (A) and its derivative FeHMP obtained after treatment with fucoidanase FFA1 from marine bacterium *Formosa algae* (B).
